# Demographics of Surgical Specialty Residency Program Directors in the United States: A Cross-sectional Analysis

**DOI:** 10.1097/AS9.0000000000000044

**Published:** 2021-03-17

**Authors:** Annika Patel, Adam Burton, Shivani Pandya, Michael Venincasa, Steven J. Gedde, Kara M. Cavuoto, Divya Sridhar, Amy Kloosterboer, Jayanth Sridhar

**Affiliations:** * Bascom Palmer Eye Institute, University of Miami Miller School of Medicine, Miami, FL; † Harlem Hospital/Columbia University Medical Center, New York, NY.

**Keywords:** residency training, Medical Education, program director

## Abstract

**Objective:**

To evaluate program director (PD) demographics, training background, and academic productivity in 11 surgical specialties.

**Summary Background Data:**

There is currently no comprehensive study comparing educational background, research output, and gender differences between PDs of surgical residencies in the United States.

**Methods:**

The Accreditation Council for Graduate Medical Education (ACGME) and the Association of American Medical Colleges (AAMC) websites were used to identify residency PDs. Age, information related to service as PD, educational background, and research output were collected utilizing online searches including Doximity, PubMed, and Scopus. The ACGME Data Resource Book was used to obtain data on the gender makeup of residents in each surgical specialty. Data collection occurred between December 14, 2019, and May 9, 2020.

**Results:**

One thousand five hundred seventy-one residency PDs across 11 surgical specialties were included. Significant differences between specialties were found with respect to PD gender, current age, age at appointment, years between residency and assignment, term duration, number of PubMed publications, and Scopus h-index. The current age (mean ± SD) ranged from 46.8 ± 8.5 years among Interventional Radiology (IR) PDs to 53.4 ± 9.1 years among Neurological Surgery (NEUROSURG) PDs. The proportion of female PDs ranged from 5.9% in NEUROSURG to 63.5% in Obstetrics and Gynecology (OB-GYN). Completion of a postresidency fellowship was least common for OB-GYN PDs at 9.1% and most common for IR PDs at 98.8%. The number (mean ± SD) of PubMed publications and Scopus h-index ranged from 13.1 ± 22.3 publications and h index 4.5 ± 5.7 among OB-GYN PDs to 112.5 ± 103.0 publications and h index 27.4 ± 16.7 among Thoracic Surgery PDs. Age and academic productivity as measured by PubMed publications and Scopus h-index were significantly lower among female PDs in multiple surgical specialties.

**Conclusions:**

There were significant variations in the PDs of surgical specialties, particularly with respect to gender and academic productivity. Efforts should be made to support and encourage greater female representation in the role of surgical residency PD.

## Abstract 2

**Mini-abstract:** A cross-sectional analysis on the program directors of surgical specialties with respect to age, gender, educational background, and academic productivity. Overall, there are significant differences among the program directors, particularly with regard to gender, and efforts should be made to increase female representation among surgical program directors.

The Accreditation Council for Graduate Medical Education (ACGME) defines the program director (PD) as the individual responsible for the oversight of an entire residency program, including aspects of administration, education, evaluation, and discipline.^1^ The qualities of PDs should ideally be a reflection of both the values of a field and the body of residents they represent. As such, by examining the differences between PDs from various residency specialties, one may gain insight into the relative weighted value of factors—such as professional experience, research output, and level of previous training—for each field.

A particularly interesting factor to analyze is gender disparities in surgical specialties. Medicine has experienced a long history of female underrepresentation, and while this trend has improved over time, there remains a large divide in higher academic positions and in surgical specialties.^2^ A recent study found a general trend of underrepresentation of females among ophthalmology residency PDs.^3^ Similarly, of the 814 plastic surgeons studied across the United States, women had less experience *(P* < 0.001), lower academic ranks *(P* < 0.001), and lower academic productivity *(P* < 0.001).^4^ Evaluation of how female PDs compare to their male counterparts in age and academic productivity in each surgical specialty would potentially identify differences between the various specialties. As such, the purpose of this study is to evaluate for differences in age, graduate and postgraduate training, academic productivity, and gender representation between the residency PDs of 11 surgical specialties across the United States.

## METHODS

This study adhered to the tenets of the Declaration of Helsinki, and Institutional Review Board (IRB) approval was waived by the University of Miami IRB. This cross-sectional study was performed using freely available online information between December 14, 2019, and May 9, 2020, selecting for ACGME accredited programs in the United States in 11 surgical subspecialties: General Surgery (GEN, 333 programs), Interventional Radiology-Integrated (IR, 86 programs), Neurological Surgery (NEUROSURG, 118 programs), Obstetrics and Gynecology (OB-GYN, 285 programs), Ophthalmology (OPH, 116 programs), Orthopedic Surgery (ORTH, 196 programs), Otolaryngology (ENT, 122 programs), Plastic Surgery-Integrated (PLAS, 81 programs), Thoracic Surgery-Integrated (THOR, 28 programs), Urology (URO, 143 programs), and Vascular Surgery-Integrated (VASC, 63 programs). Programs without clearly presented data on PDs were excluded (39 programs total).

The ACGME website (https://www.acgme.org/) and Association of American Medical Colleges (AAMC) residency search (https://www.aamc.org/) were used to obtain information on PD name, appointment year, gender, and academic degrees. Information on medical school, residency, fellowship attendance, and graduation year was collected from Doximity (https://www.doximity.com/). Physician age was found on Healthgrades (https://www.healthgrades.com/). PubMed (https://pubmed.ncbi.nlm.nih.gov/) and Scopus (https://www.scopus.com/) were used to determine the number of PubMed publications and the Scopus h-index of each program director. The h-index is an indicator of academic productivity that is measured by the number of papers, *h*, with *≥h* citations.^5^ The ACGME Data Resource Book was used to obtain data on the gender makeup of residents in each surgical specialty.^6^

The data were recorded and analyzed using Excel 2018 (Microsoft, Inc., Redmond, WA). Statistical analyses were performed using SPSS version 25.0 (IBM, Armonk, NY) and included 2 sample t-test, χ^2^ test, Kruskal-Wallis test, Pearson likelihood ratio, and multivariate regression. A *P* value <0.05 was considered statistically significant.

## RESULTS

A total of 1571 residency PDs from the 11 surgical subspecialties were included for analysis. In total, 39 PDs from 39 programs were excluded from analysis due to lack of clearly presented data on PDs: 5 GEN, 3 IR, 1 NEUROSURG, 3 OB-GYN, 9 OPH, 8 ORTH, 4 ENT, 1 PLAS, 2 THOR, 2 URO, and 1 VASC. Different *n* values were used for each parameter based on how many programs for which the information was available (Appendix Table 1).

### Current Age Demographics

When comparing all specialties together, there was a significant difference in PD age (*P* < 0.001). IR PDs generally had the youngest current age, with mean ± SD (range) age of 46.8 ± 8.5 (34.0–74.0) years, and NEUROSURG PDs were the oldest at 53.4 ± 9.1 (38.0–80.0) years (Table 1). IR PDs were significantly younger than URO, PLAS, ORTH, GEN, and NEUROSURG PDs, and OB-GYN PDs were significantly younger than GEN PDs (all *P* < 0.05).

**Table 1.** Residency Program Director Descriptive Statistics by Surgical Specialty

	GEN	IR	NEUROSURG	OB-GYN	OPH	ORTH	ENT	PLAS	THOR	URO	VASC
**Program count**	333	86	118	285	116	196	122	81	28	143	63
**Mean current age (years**)	52.9 ± 8.7	46.8 ± 8.5	53.4 ± 9.1	50.0 ± 8.6	50.0 ± 10.3	52.5 ± 9.0	50.4 ± 8.4	52.3 ± 9.3	52.1 ± 7.0	51.9 ± 9.3	50.1 ± 6.8
**Mean age at appointment (years**)	46.9 ± 8.2	44.3 ± 8.1	47.6 ± 7.8	43.9 ± 6.7	42.9 ± 9.8	44.8 ± 7.4	43.4 ± 7.2	47.2 ± 8.1	47.5 ± 6.9	44.6 ± 7.5	45.1 ± 6.4
**Mean time post residency to assignment (years**)	14.2 ± 8.6	12.5 ± 8.5	13.8 ± 7.8	12.4 ± 6.7	10.7 ± 9.1	12.6 ± 7.1	10.3 ± 7.1	12.5 ± 7.4	14.5 ± 6.7	11.8 ± 7.1	13.2 ± 6.9
**Mean term duration (years**)	5.6 ± 5.0	2.7 ± 1.2	5.8 ± 5.1	5.8 ± 5.8	7.6 ± 5.5	7.2 ± 6.3	6.2 ± 5.2	4.8 ± 3.9	4.3 ± 2.8	6.6 ± 6.6	4.8 ± 3.4
**Mean number of PubMed publications**	35.9 ± 88.3	23.7 ± 30.4	74.4 ± 81.3	13.1 ± 22.3	17.6 ± 21.2	34.5 ± 54.6	35.3 ± 45.5	46.0 ± 45.3	112.5 ± 103.0	71.0 ± 136.2	71.2 ± 65.8
**Mean Scopus h-index**	12.9 ± 13.0	7.5 ± 9.5	23.6 ± 16.5	4.5 ± 5.7	8.7 ± 7.6	9.7 ± 8.6	10.3 ± 9.5	12.4 ± 8.7	27.4 ± 16.7	16.7 ± 15.3	19.5 ± 12.1
**Female PDs (count, %**)	68 (20.5%)	10 (11.6%)	7 (5.9%)	181 (63.5%)	32 (27.6%)	19 (9.7%)	36 (29.5%)	17 (21.0%)	4 (14.3%)	24 (16.8%)	8 (12.7%)
**PD of same program as residency training (count, %**)	74 (25.3%)	21 (24.7%)	28 (27.5%)	76 (32.1%)	30 (25.9%)	81 (41.3%)	41 (33.6%)	27 (33.8%)	7 (25.0%)	34 (23.9%)	17 (27.0%)
**International Medical School graduate (count, %**)	37 (11.1%)	11 (12.8%)	13 (11%)	14 (5.3%)	8 (6.9%)	10 (5.1%)	6 (5.0%)	9 (11.1%)	1 (3.6%)	12 (8.4%)	10 (15.9%)
**DO degree (count, %**)	27 (8.4%)	3 (3.5%)	3 (2.5%)	36 (12.6%)	3 (2.6%)	24 (12.2%)	11 (9.0%)	0 (0.0%)	0 (0.0%)	6 (4.2%)	0 (0.0%)
**Additional degree (count, %**)	47 (14.1%)	7 (8.1%)	23 (19.5%)	51 (17.9%)	23 (19.8%)	16 (8.2%)	20 (16.4%)	15 (18.5%)	4 (14.3%)	27 (18.9%)	8 (12.7%)
**MS, MSc (count, %**)	10 (3.0%)	1 (1.2%)	3 (2.5%)	17 (6.0%)	8 (6.9%)	1 (0.5%)	6 (4.9%)	3 (3.7%)	1 (3.6%)	9 (6.3%)	5 (7.9%)
**PhD (count, %**)	6 (1.8%)	2 (2.3%)	17 (14.4%)	5 (1.8%)	7 (6.0%)	2 (1.0%)	3 (2.5%)	4 (4.9%)	2 (7.1%)	5 (3.5%)	0 (0.0%)
**MPH (count, %**)	12 (3.6%)	1 (1.2%)	1 (0.8%)	16 (5.6%)	4 (3.4%)	4 (2.0%)	6 (4.9%)	1 (1.2%)	0 (0.0%)	3 (2.1%)	1 (1.6%)
**MBA (count, %**)	12 (3.6%)	0 (0.0%)	2 (1.7%)	1 (0.4%)	3 (2.6%)	5 (2.6%)	2 (1.6%)	2 (2.5%)	0 (0.0%)	7 (4.9%)	0 (0.0%)
**Fellowship completion (count, %**)	138 (41.4%)	85 (98.8%)	24 (20.3%)	26 (9.1%)	90 (77.6%)	166 (84.7%)	84 (68.9%)	63 (77.8%)	27 (96.4%)	105 (73.4%)	61 (96.8%)

All means shown ± one standard deviation.

DO, doctor of osteopathic medicine; ENT, otolaryngology; GEN, general surgery; IR, interventional radiology; MBA, masters in business administration degree.; MPH, masters in public health degree; MS, masters degree; MSc, masters of science degree; NEUROSURG, neurosurgery; OB-GYN, obstetrics and gynecology; OPH, ophthalmology; ORTH, orthopedic surgery; PD, Program director; PhD, doctor of philosophy degree; PLAST, plastic and reconstructive surgery; THOR, thoracic surgery; URO, urologic surgery; VASC, vascular surgery.

### Age of Appointment

When comparing all specialties together, there was a significant difference between the age at appointment of surgical specialty PDs (*P* < 0.001). OPH PDs were found to be the youngest at appointment, with a mean ± SD (range) of 42.9 ± 9.8 (31.0–72.0) years, and NEUROSURG PDs were the oldest at appointment, at 47.6 ± 7.8 (36.0–79.0) years (Table 2). OPH PDs were significantly younger at appointment than GEN, PLAS, NEUROSURG, and THOR PDs. NEUROSURG PDs were significantly older at appointment than ENT, IR, and OB-GYN PDs. GEN PDs were significantly older at appointment than ENT and OB-GYN PDs (all *P* < 0.05).

**Table 2.** Most Commonly Attended Programs by Residency Program Directors in Each Surgical Specialty

	Most Common Medical Schools Attended		Most Common Residency Program Attended		Most Common Fellowship Program Attended	
		Count (%)*		Count (%)*		Count (%)*
**GEN**	Sidney Kimmel Medical College	9 (2.8%)	Massachusetts General Hospital	6 (2.0%)	Baylor University Medical Center	5 (3.6%)
	University of Texas Medical School	9 (2.8%)	University of Illinois College of Medicine	6 (2.0%)	Jackson Memorial Hospital - Jackson Health System	5 (3.6%)
	Wayne State University School of Medicine	9 (2.8%)			Mayo School of Graduate Medical Education	5 (3.6%)
					University of Washington	5 (3.6%)
**IR**	Boston University School of Medicine	5 (5.8%)	North Shore-Long Island Jewish Health System	4 (4.7%)	McGaw Medical Center of Northwestern University	7 (8.2%)
	Georgetown University School of Medicine	4 (4.7%)	University of Illinois College of Medicine at Chicago	3 (3.5%)	Brigham and Women’s Hospital	5 (5.9%)
	State University of New York Upstate Medical University	3 (3.5%)	University of Rochester	3 (3.5%)		
	Tulane University School of Medicine	3 (3.5%)				
**NEUROSURG**	Columbia University College of Physicians & Surgeons	6 (5.6%)	UCLA Medical Center	4 (4.0%)	Barrow Neurological Institute	2 (8.3%)
	Georgetown University School of Medicine	4 (3.7%)	University of Pittsburgh Medical Center	4 (4.0%)	Johns Hopkins University School of Medicine	2 (8.3%)
	Rutgers Robert Wood Johnson Medical School	4 (3.7%)				
**OB-GYN**	University of Texas Medical School	7 (2.7%)	Albert Einstein College of Medicine	6 (2.5%)	University of Pennsylvania Health System	2 (7.7%)
	Michigan State University College of Osteopathic Medicine	6 (2.3%)	NYU School of Medicine	5 (2.1%)	Yale University School of Medicine	2 (7.7%)
			University of Texas Southwestern Medical School	5 (2.1%)		
**OPH**	Baylor College Of Medicine	4 (3.4%)	Wayne State University	4 (3.4%)	Bascom Palmer Eye Institute	6 (6.7%)
	Harvard Medical School	4 (3.4%)			Wills Eye Hospital	6 (6.7%)
	University of Pennsylvania School of Medicine	4 (3.4%)			University of Utah	4 (4.4%)
**ORTH**	Philadelphia College of Osteopathic Medicine	6 (3.1%)	Mount Sinai School of Medicine	5 (2.6%)	Massachussetts General Hospital	9 (5.4%)
	Sidney Kimmel Medical College	6 (3.1%)			Hospital for Special Surgery	5 (3.0%)
	University of Illinois College of Medicine	6 (3.1%)				
**ENT**	Eastern Virginia Medical School	5 (4.1%)	Mount Sinai School of Medicine	4 (3.3%)	Massachusetts Eye & Ear Infirmary	6 (7.1%)
	Sidney Kimmel Medical College	5 (4.1%)	UCLA Medical Center	4 (3.3%)	Medical College of Georgia	5 (6.0%)
	Ohio State University	4 (3.3%)	University of Michigan Hospitals and Health Centers	4 (3.3%)		
**PLAS**	Harvard Medical School	4 (4.9%)	University of Pittsburgh Medical Center	7 (8.8%)	University of Pittsburgh Medical Center	7 (11.1%)
	Johns Hopkins University School of Medicine	4 (4.9%)	Wake Forest University	5 (6.3%)	Johns Hopkins University	4 (6.3%)
	Perelman School of Medicine at the University of Pennsylvania	4 (4.9%)	Mayo School of Graduate Medical Education	5 (6.3%)	NYU School of Medicine	4 (6.3%)
**THOR**	Columbia University College of Physicians & Surgeons	3 (10.7%)	Massachussetts General Hospital	3 (10.7%)	University of Michigan Hospitals	4 (14.8%)
	Johns Hopkins University School of Medicine	3 (10.7%)			Duke University Hospital	3 (11.1%)
	University of Maryland School of Medicine	3 (10.7%)				
**URO**	Columbia University College of Physicians & Surgeons	6 (4.2%)	UCLA Medical Center	6 (4.2%)	Memorial Sloan Kettering Cancer Center	9 (8.6%)
	NYU School of Medicine	6 (4.2%)	Cleveland Clinic Foundation	5 (3.5%)	UCSF	7 (6.7%)
	Baylor College of Medicine	5 (3.5%)			Cleveland Clinic Foundation	7 (6.7%)
	UCLA Medical School	5 (3.5%)				
**VASC**	NYU School of Medicine	3 (4.8%)	New York Medical College	4 (6.3%)	University of Michigan Hospitals and Health Centers	5 (8.2%)
	Southern Illinois University School of Medicine	3 (4.8%)	NYU School of Medicine	3 (4.8%)	Washington University School of Medicine- St Louis	4 (6.6%)
					Massachussetts General Hospital	4 (6.6%)

*Out of those for which program attendance information is available.

ENT, otolaryngology; GEN, general surgery; IR, interventional radiology; NEUROSURG, neurosurgery; OB-GYN, obstetrics and gynecology; OPH, ophthalmology; ORTH, orthopedic surgery; PLAST, plastic and reconstructive surgery; THOR, thoracic surgery; URO, urologic surgery; VASC, vascular surgery.

### Time to Appointment After Completing Training

When comparing all specialties together, the number of years postresidency graduation to PD assignment was significantly different (*P* < 0.001), independent of whether a fellowship was completed. ENT PDs were found to have the shortest average time between completion of postresidency training and appointment as PD (mean ± SD, 10.3 ± 7.1; median, 9.0; range, 1.0–38.0 years). In contrast, THOR PDs were found to have the longest average interim time (mean ± SD, 14.5 ± 6.7; median, 13.5; range, 3.0–32.0 years) (Table 1). OPH PDs had significantly shorter time to appointment when compared with ORTH, OB-GYN, VASC, NEUROSURG, GEN, and THOR PDs (all *P* < 0.05). In addition, ENT PDs were found to have significantly shorter time to appointment than ORTH, OB-GYN, NEURO, GEN, and THOR PDs (all *P* < 0.05).

### Length of Appointment

When comparing all specialties, the term duration was significantly different (*H* = 70.013, *P* < 0.001), with a significantly shorter term duration of IR PDs compared with OB-GYN, GEN, NEUROSURG, URO, ENT, ORTHO, and OPH PDs, as well as a significantly long-term duration for OPH PDs compared with PLAS, OB-GYN, and GEN PDs (all *P* < 0.05). IR PDs were found to have the shortest average current term (mean ± SD, 2.7 ± 1.2; median, 3.0; range 1.0–4.0 years), and OPH PDs were found to have the longest average current term (mean ± SD, 7.6 ± 5.5; median, 6; range 1.0–24.0 years) (Table 1).

### Graduate/Postgraduate Training

The majority of PDs received their medical school training in the United States, with THOR having the lowest percentage of internationally trained PDs (3.6%), and VASC having the highest percentage of internationally trained PDs (15.9%) (Table 1). For international trainees, India was the most common country of medical school training for GEN, IR, THOR, URO, and VASC PDs, and Canada was the most common for NEUROSURG, ORTH, and ENT PDs.

The majority of PDs across all surgical specialties had an MD degree. All PLAS, THOR, and VASC PDs had an MD degree, while 12.6% of OB-GYN PDs had a DO degree. OPH had the greatest percentage of PDs with an additional degree (19.8%), and IR had the lowest percentage (8.1%). The most common additional degrees among all surgical specialty PDs were MS (or its international equivalent, MSc), PhD, MPH, and MBA (Table 1).

Table 2 displays the most common medical schools, residency programs, and fellowship programs attended by the PDs of each surgical specialty. The percentage of PDs that completed a fellowship varied greatly among the surgical specialties, with IR having the highest (98.8%), and OB-GYN having the lowest (9.1%). The percentage of PDs that served as PD for their original residency program varied greatly, with ORTH having the greatest percentage (41.3%), and URO having the lowest percentage (23.9%).

Multivariate regression analysis revealed that fellowship completion across all surgical PDs was associated with a younger current age (*P* < 0.001) and fewer years postresidency graduation to PD assignment (*P* < 0.001). When specialties were evaluated separately, fellowship completion was associated with a younger current age for PDs in IR, ORTH, PLAS, and URO, but an older current age for THOR and VASC (all *P* < 0.05). Fellowship completion was associated with fewer years between residency and PD assignment for IR and ORTH (both *P* < 0.05). A Pearson Likelihood Test showed a significant relationship between specialty and fellowship completion rate (*Χ*^2^ = 595.6, df = 10, *P* < 0.001).

### Gender

Of the 11 surgical specialties, only OB-GYN had a majority female representation (63.5%). NEUROSURG had the fewest female PDs (5.9%). When compared with the gender breakdown of residents in each surgical specialty as reported by ACGME, females made up a lower percentage of program directors in all 11 surgical specialties (Table 3). This difference was found to be statistically significant for GEN, NEUROSURG, OB-GYN, OPH, ORTH, PLAS, URO, and VASC (all *P* < 0.05). In addition, female PDs were on average younger than male PDs for all 11 specialties. This difference was statistically significant for GEN, OB-GYN, ORTH, ENT, and URO (all *P* < 0.05).

**Table 3.** Gender Differences Among Surgical Specialties

	Female Composition			Age by Gender			PubMed Publications by Gender			Scopus h-index by Gender		
	**Female PDs (count, %**)	**Female Residents (count, %**)	***P*** *	Mean Female Age	Mean Male Age	***P *** ^**b**^	Mean Female Publications	Mean Male Publications	***P *** ^**b**^	Mean Female h-index	Mean Male h-index	***P *** ^**b**^
**GEN**	68 (20.5%)	3443 (38.9%)	***P*** < **0.001**	48.0 ± 8.1	54.0 ± 8.4	***P*** < **0.001**	26.5 ± 51.1	38.4 ± 95.6	*P* = 0.170	9.3 ± 11.0	13.8 ± 13.3	***P*** = **0.005**
**IR**	10 (11.6%)	45 (18.3%)	*P* = 0.152	45.8 ± 7.0	46.9 ± 8.8	*P* = 0.643	6.8 ± 6.7	25.9 ± 31.8	***P*** < **0.001**	4.4 ± 3.1	7.9 ± 10.0	***P*** = **0.025**
**NEUROSURG**	7 (5.9%)	253 (17.3%)	***P*** = **0.001**	46.8 ± 6.9	53.8 ± 9.1	*P* = 0.057	61.6 ± 75.4	75.2 ± 82.2	*P* = 0.660	17.4 ± 14.0	23.9 ± 16.7	*P* = 0.275
**OB-GYN**	181 (63.5%)	4559 (81.8%)	***P*** < **0.001**	47.7 ± 7.0	53.9 ± 9.7	***P*** < **0.001**	12.0 ± 19.9	15.1 ± 26.3	*P* = 0.348	4.1 ± 5.2	5.3 ± 6.4	*P* = 0.148
**OPH**	32 (27.6%)	575 (39.0%)	***P*** = **0.013**	47.4 ± 9.8	51.0 ±10.4	*P* = 0.112	13.5 ± 23.1	19.2 ± 20.5	*P* = 0.240	5.4 ± 4.0	9.8 ± 8.3	***P*** = **0.001**
**ORTH**	19 (9.7%)	606 (14.7%)	***P*** < **0.001**	48.0 ± 6.6	53.0 ± 9.1	***P*** = **0.009**	29.6 ± 27.2	35.0 ± 56.8	*P* = 0.570	9.8 ± 6.6	9.7 ± 8.8	*P* = 0.952
**ENT**	36 (29.5%)	589 (35.7%)	*P* = 0.141	47.7 ± 7.9	51.5 ± 8.5	***P*** = **0.044**	30.4 ± 28.7	37.1 ± 50.9	*P* = 0.449	9.1 ± 7.0	10.8 ± 10.4	*P* = 0.311
**PLAS**	17 (21%)	366 (40.6%)	***P*** < **0.001**	49.3 ± 7.9	53.1 ± 9.7	*P* = 0.148	26.0 ± 24.0	50.8 ± 48.7	***P*** = **0.037**	8.2 ± 6.3	13.5 ± 9.0	***P*** = **0.011**
**THOR**	4 (14.3%)	55 (29.1%)	*P* = 0.094	50.5 ± 12.7	52.4 ± 6.1	*P* = 0.791	37.0 ± 24.5	126.6 ± 109.5	***P*** = **0.010**	14.5 ± 7.9	29.6 ± 17.3	***P*** = **0.020**
**URO**	24 (16.8%)	333 (25.0%)	***P*** = **0.029**	46.3 ± 7.0	52.8 ± 9.4	***P*** = **0.003**	49.5 ± 75.9	74.7 ± 144.9	*P* = 0.356	10.0 ± 9.4	18.0 ± 15.9	***P*** = **0.002**
**VASC**	8 (12.7%)	96 (32.9%)	***P*** = **0.001**	48.8 ± 6.2	50.3 ± 6.9	*P* = 0.604	57.7 ± 70.4	72.2 ± 67.0	*P* = 0.759	16.0 ± 10.7	20.0 ± 12.4	*P* = 0.352

All means shown ± one standard deviation. Bolded *P* values reflect statistical significance (*P *<* *0.05).

*Pearson chi-square test; ^b^two-sample t test.

ENT, otolaryngology; GEN, general surgery; IR, interventional radiology; NEUROSURG, neurosurgery; OB-GYN, obstetrics and gynecology; OPH, ophthalmology; ORTH, orthopedic surgery; PD, residency program director; PLAST, plastic and reconstructive surgery; THOR, thoracic surgery; URO, urologic surgery; VASC, vascular surgery.

### Academic Productivity

THOR PDs had the greatest average number of PubMed publications (mean ± SD, 112.5 ± 103.0 publications) as well as the highest Scopus h-index (mean ± SD, 27.4 ± 16.7). OB-GYN PDs had the fewest average PubMed publications (mean ± SD, 13.1 ± 22.3) and the lowest Scopus h-index (mean ± SD, 4.5 ± 5.7) (Table 1). There was a significant difference in the number of PubMed publications between specialties, as well as in Scopus h-index between specialties (both *P* < 0.001) (Table 4). When multivariate regression was utilized to control for current age, history of fellowship training, time since residency graduation, and gender, older current age was associated with significantly lower numbers of PubMed publications for NEUROSURG, whereas longer time since residency was associated with significantly higher number of publications (both *P* < 0.05). Older current age was associated with significantly higher Scopus-h scores for OB-GYN and significantly lower scores for NEUROSURG and THOR, and increasing time since residency was associated with significantly higher scores for NEUROSURG, ENT, and THOR and significantly lower scores for OB-GYN (all *P* < 0.05).

**Table 4.** Post Hoc Dunn-Bonferroni Test to Determine Interspecialty Differences in Research Output Measures

	**PubMed Publications (mean**)		Significance* (*P*)	**Scopus h-Index (mean**)		Significance* (*P*)
**Highest**	THOR (112.5 ± 79.0)	**Compared To:**		THOR (27.4 ± 16.7)	**Compared To:**	
		GEN (35.9 ± 88.3)	**< 0.001**		GEN (12.9 ± 13.0)	**< 0.001**
		IR (23.7 ± 30.4)	**< 0.001**		IR (7.5 ± 9.5)	**< 0.001**
		NEUROSURG (74.4 ± 81.3)	1.000		NEUROSURG (23.6 ± 16.5)	1.000
		OB-GYN (13.1 ± 22.3)	**< 0.001**		OB-GYN (4.5 ± 5.7)	**< 0.001**
		OPH (17.6 ± 21.2)	**< 0.001**		OPH (8.7 ± 7.6)	**< 0.001**
		ORTH (34.5 ± 54.6)	**0.006**		ORTH (9.7 ± 8.6)	**< 0.001**
		ENT (35.3 ± 45.5)	**0.021**		ENT (10.3 ± 9.5)	**< 0.001**
		PLAS (46.0 ± 45.3)	1.000		PLAS (12.4 ± 8.7)	**0.025**
		URO (71.0 ± 136.2)	1.000		URO (16.7 ± 15.3)	0.152
		VASC (71.2 ± 65.8)	1.000		VASC (19.5 ± 12.1)	1.000
**Lowest**	OB-GYN (13.1 ± 22.3)	**Compared To:**		OB-GYN (4.5 ± 5.7)	**Compared To:**	
		GEN (35.9 ± 88.3)	**< 0.001**		GEN (12.9 ± 13.0)	**< 0.001**
		IR (23.7 ± 30.4)	**0.009**		IR (7.5 ± 9.5)	0.411
		NEUROSURG (74.4 ± 81.3)	**< 0.001**		NEUROSURG (23.6 ± 16.5)	**< 0.001**
		OPH (17.6 ± 21.2)	1.000		OPH (8.7 ± 7.6)	**< 0.001**
		ORTH (34.5 ± 54.6)	**< 0.001**		ORTH (9.7 ± 8.6)	**< 0.001**
		ENT (35.3 ± 45.5)	**< 0.001**		ENT (10.3 ± 9.5)	**< 0.001**
		PLAS (46.0 ± 45.3)	**< 0.001**		PLAS (12.4 ± 8.7)	**< 0.001**
		THOR (112.5 ± 79.0)	**< 0.001**		THOR (27.4 ± 16.7	**< 0.001**
		URO (71.0 ± 136.2)	**< 0.001**		URO (16.7 ± 15.3)	**< 0.001**
		VASC (71.2 ± 65.8)	**< 0.001**		VASC (19.5 ± 12.1)	**< 0.001**

All means shown ± one standard deviation. Bolded *P* values reflect statistical significance (*P* < 0.05).

*Significance calculated using Post-Hoc Dunn-Bonferroni test.

ENT, otolaryngology; GEN, general surgery; IR, interventional radiology; NEUROSURG, neurosurgery; OB-GYN, obstetrics and gynecology; OPH, ophthalmology; ORTH, orthopedic surgery; PLAST, plastic and reconstructive surgery; THOR, thoracic surgery; URO, urologic surgery; VASC, vascular surgery.

When all surgical specialties were considered together, only gender showed significant association with PubMed publications (*P* = 0.003) and Scopus-h scores (*P* < 0.001). The number of PubMed publications was found to be significantly different between males and female PDs in IR, PLAS, and THOR (all *P* < 0.05). The Scopus h-indices were found to be significantly different between males and female PDs in GEN, IR, OPH, PLAS, THOR, and URO (all *P* < 0.05) (Table 3). When multivariate regression was utilized to control for current age, history of fellowship training, and time since residency graduation, female gender remained associated with significantly lower numbers of PubMed publications and Scopus h-indices for PDs (*P* < 0.0001 for both) when all surgical specialties were compared together but not for each individual specialty.

## DISCUSSION

The aim of this study was to evaluate differences in age, graduate and postgraduate training, gender, and scholarly productivity between PDs across 11 surgical specialties. Inter-specialty differences were found in all areas analyzed, which may provide insight into the relative values of the individual fields with respect to expected scholarly output and other demographics for residency PDs.

The most revealing findings of this study were the PD gender differences for each surgical specialty. Ten of the 11 specialties analyzed (all specialties except OB-GYN) had less than 50% female PDs, but more telling is that the percentage of female PDs was lower than the percentage of female residents across all 11 surgical specialties, with a statistically significant difference in 8 out of 11 specialties. A report by the AAMC demonstrated that there is less female representation in medicine as one progresses in rank, with women making up 51% of medical school applicants, 48% of medical school graduates, 46% of residents, 41% of faculty, 29% of division chiefs, 18% of department chairs, and 18% of deans.^2^ Studies show a steady increase in female representation in medicine over recent years^2^, although this increase appears to vary when stratified by specialty. For example, the rate of increase of women in ORTH has been markedly lower than other surgical fields.^7,8^ Despite this increase, a recent publication demonstrated that over a 35–year-period women in academic medical centers were less likely than men to be promoted or to achieve the rank of department chair with no change in trend over that time period.^9^ To our knowledge, there is no recent data on the trend of female representation in the PD role specifically, and further research into trends over time may provide useful information. Age was found to be significantly different between male and female PDs within many surgical specialties, with female PDs generally being younger than their male counterparts. This is consistent with results of a study done on GEN PDs that found that male and female PDs were often appointed a similar amount of time after residency graduation, but men tended to hold their position for longer.^10^ However, the younger current ages of female PDs compared with male PDs may also be an encouraging indication of increased appointment of female PDs in recent years, as the more recent appointments are likely to also be the younger appointments. Given the lag between graduation from residency and appointment as PD, some of these differences between resident and PD demographics are likely due to the increasing number of women entering surgical subspecialties in recent years, and perhaps lend hope for the future as an increasing representation of women in medicine trickles up to leadership positions.

Academic productivity, measured by PubMed publications and Scopus h-index, is not a requirement for a PD, but was included in this study to offer another data point to compare PDs within a specialty. Research output was generally lower in female PDs compared to male PDs in all surgical specialties but was significantly lower for IR, PLAS, and THOR. When controlling for age, fellowship status, and time since residency, this relationship remained when all specialties were compared together. While this may suggest lower academic productivity among the female PDs, it may also illustrate an effort to appoint PDs on the basis of intangible and important factors other than academic productivity, such as communication and leadership skills. To evaluate whether the lower academic productivity seen in current female PDs is indicative of lower interest and involvement in research overall, future studies could compare the longitudinal distribution of scholarly output, as a study done on physicians at the Mayo Clinic found that women produced significantly less research than males in the first 27 years of service, but significantly more research after this time period.^11^ If this trend also applies to surgical specialty PDs, it could indicate that female PDs do not have less academic potential than their male counterparts but instead may be altered by life events that disproportionately affect female physicians, such as childbirth. A survey completed by 347 female surgeons with 452 pregnancies revealed that 63.6% felt that their work schedule impacted their health or the health of their unborn child in a negative way, and 66.8% wished for greater mentorship in how to integrate pregnancy and motherhood into a surgical career.^12^ This suggests a greater need for surgical residency programs, and medicine as a whole, to provide support for female physicians to allow them to reach their academic potential. This is especially true considering that a prior study demonstrated that female medical school faculty do not advance academically as rapidly nor are compensated as well as their male counterparts of similar professional rank, and that this disparity worsens at higher academic ranks.^13^

Notably, the difference between male and female research output was smallest in OB-GYN, which has the largest female representation, and largest in THOR, which has among the lowest female representation. This discrepancy suggests that in fields that have prominent inclusion of females such as OB-GYN, academic productivity may be used to compare counterparts on a more equal basis than fields with significantly lower female inclusion such as THOR, which may support appointment of female PDs who do not have the same “on paper” academic productivity as their male counterparts. This discrepancy is important for evaluating the priorities of different surgical fields. Specialties such as THOR that have historically included more male physicians may be prioritizing the inclusion of women in PD positions despite lower research output, perhaps due to increasing importance being placed on other leadership qualities over academic productivity.

The ACGME’s requirements for PDs include at least 3 years of academic or administrative experience before appointment to allow for the development of leadership and professional skills, which may be bypassed if the candidate is shown to fulfill a community’s needs.^1^ We did not capture this exact data for individual PDs given that the exact definition of what administrative experience constitutes may vary. Still, surgical specialties all had an average of 10 years or more between residency completion and PD appointments, suggesting likely compliance. The ACGME also encourages longevity of the PD term to master the necessary skills to best carry out the PD role.^1^ The surgical specialties varied in the length of their current term, with OPH having the longest average current term and IR the shortest. However, this is almost certainly confounded by the American Board of Medical Specialties naming IR a separate specialty in 2012, allowing IR to transition from a fellowship to a dedicated residency program.^14^ As such, most IR residency programs are less than four years old, and PD terms would have to be shorter. While there are some potential benefits to PD turnover, including injection of innovation and new educational ideas, this suggests that some surgical subspecialties with long-term duration, including OPH, may be providing resources and mentorship to PDs that encourage positional longevity.

The differences in age demographics and educational backgrounds of PDs suggest variation in the values deemed important for different surgical fields. The average current age of PDs across specialties ranged from 46.8 to 53.4 years, which is quite narrow and can likely be partially explained by differences in length of training. The more revealing aspect of evaluating age discrepancies lies in the time between residency training and PD assignment. For example, NEUROSURG PDs were found to be the oldest current PDs, oldest at appointment, and among the top 3 for number of years between completion of residency training and appointment compared with other surgical specialties. While this may be a factor of the longer duration of residency training in NEUROSURG, it is also possible that the NEUROSURG field values experience in their PDs or have lower turnover of these leadership positions. In contrast, OPH PDs were found to have the 2nd youngest current age, the youngest age at appointment, and the shortest duration before appointment, suggesting that the field may value recent training. While it is important to take into account that the NEUROSURG residency training is 7 years and OPH residency training is 3 years after an internship year, this could explain the discrepancies in current age and age at appointment but should not impact the number of years between residency training and PD assignment. These findings suggest NEUROSURG may value PDs with more experience in the work field, and OPH may value recent training.

For all 11 surgical specialties, the majority of PDs had an MD degree received from a medical school in the United States, although the percentage of PDs with a DO degree and medical training outside of the United States varied from specialty to specialty. The percentage of PDs who completed fellowships, as well as the percentage of PDs with an additional degree besides their medical degree, also varied greatly from specialty to specialty. For example, IR (98.8% fellowship completion), THOR (96.4% fellowship completion), and VASC (96.8% fellowship completion) all had close to 100% of their PDs complete a fellowship, which can be attributed to these fields’ histories of requiring separate fellowships after a more general residency such as Diagnostic Radiology before IR, or GEN before THOR and VASC. In contrast, 9.1% of OB-GYN PDs have completed a fellowship. Interestingly, fellowship completion was associated with a younger current age and fewer years postresidency completion to PD assignment, suggesting that attaining a fellowship may be a fast-track for obtaining higher ranks. This association was significant for IR and ORTH for both measures, and PLAS and URO for current age. THOR and VASC showed the opposite relationship, with fellowship completion being associated with an older current age.

Research output in the form of both numbers of PubMed publications and Scopus h-index was found to have high variability within each surgical specialty, as well as between surgical specialties. The variability may suggest different relative importance placed on scholarly productivity between fields; regardless, extensive scholarly productivity is not part of the ACGME’s requirement for residency PDs and likely does not correlate with success in the PD role. Variation between PDs of different surgical specialties can be seen by the stark difference in PubMed publications and Scopus h-index between THOR (112.5 ± 79.0 publications; h-index, 27.4 ± 16.7) and OB-GYN (13.1 ± 22.3 publications, h-index, 4.5 ± 5.7). A study on academic productivity of physicians in general surgery and surgical specialties similarly found the highest h-indices in thoracic surgeons and surgical oncologists, perhaps reflective of the relative importance placed on academic productivity, as dedicated research time is often required to match into fellowships in these fields.^15^

A major limitation of this study was the reliance on freely available online information with cross-sectional analysis limited to the 2019–2020 academic year. Theoretically, PD turnover during the academic year could have altered the results if it was not reflected on the ACGME website. Statistics such as percentage with a completed fellowship may be underestimates of the actual value due to failure of websites to provide updated information. Specific data of other fields, such as current age, were also not always available, meaning the statistics may not be representative of the entire PD body of each surgical specialty (see Appendix Table 1). Data collection for this study took place over a span of almost 5 months, which serves as a limitation due to the possibility of changes in PD appointments or ages within this span. In addition, information about PubMed publications and Scopus h-index was retrieved by entering the PD’s first and last name, and when possible, middle initial into the search engine. The possibility of authors with the same name may mean inaccurate numbers for the academic productivity values, as well as authors who may have changed name due to marriage or other life circumstance.

Another limitation to consider is that the differences between specialties in PD current age or years postresidency graduation to PD appointment was found to be significantly associated with whether or not a fellowship was completed. For these specialties, differences in current age and years between residency appointment and PD assignment can be due to time taken for fellowship completion and other factors rather than due to the differences in values between the fields. Specifically, IR and OB-GYN PDs were found to be significantly younger than GEN PDs. OPH and ENT PDs were found to have a significantly shorter time between residency completion and PD assignment than ORTH, OB-GYN, NEURO, GEN, and THOR PDs. However, the rates of fellowship completion were found to be significantly different between specialties. While IR and OB-GYN both have younger PDs, IR had among the highest fellowship completion rate, and OB-GYN the lowest. Similarly, OB-GYN, NEURO, and THOR were all found to have a longer time period between residency graduation and appointment to PD, but much fewer OB-GYN and NEURO PDs completed fellowship compared with THOR. Thus, the differences in current age and time between residency graduation and fellowship completion may be affected by pursuit of a fellowship.

In summary, differences in age demographics, training, gender representation, and scholarly productivity were seen among PDs of surgical specialties. Results from our study indicate that female PDs tended to be younger and may suggest an upward trend in female representation. Still, the underrepresentation of women as PDs relative to the percentage of female surgical residents suggests that efforts must be made to encourage women leadership in surgical specialty residency programs. Potential solutions include but are not limited to increased support in terms of paid leave and resources for women pre- and postpartum to make balancing work and family more manageable, organizing departmental meetings to be held virtually and at times more conducive to attendance by physicians who bear child-care responsibilities, and formal mentorship programs designed to foster interest in residency leadership in younger female attendings. Not only will increased female leadership make the PD pool more representative of trainees, it will also provide role models and mentors who can encourage the continued inclusion of women in surgical fields as a whole.

## Appendix

**Appendix Table 1.** Parameter Ns for Residency Program Director Characteristics

Specialty	GEN	IR	NEUROSURG	OB-GYN	OPH	ORTH	ENT	PLAS	THOR	URO	VASC
**Actual total program count**	333	86	118	285	116	196	122	81	28	143	63
**Current age***	294	83	101	242	102	171	94	70	26	117	55
**Age at appointment***	294	82	102	241	102	171	94	70	26	117	55
**Years post residency to assignment***	291	81	101	233	116	193	121	80	28	140	61
**Term duration***	332	83	118	285	116	196	122	81	28	143	63
**Gender***	332	86	118	285	116	196	122	81	28	143	63
**Same program as residency training***	292	85	102	237	116	196	122	80	28	142	63
**Medical school attended***	321	86	108	256	116	196	122	81	28	143	63
**Medical degree***	322	86	118	285	116	196	122	80	28	143	63
**Additional degree****	47	7	23	51	23	16	20	15	4	27	8
**Residency attended***	293	85	101	237	116	196	122	80	28	142	63
**Fellowship attended***	138	85	24	26	90	166	84	63	27	105	61
**Number of PubMed Publications***	327	86	118	236	108	134	84	46	19	89	44
**Scopus h-index***	327	85	117	237	102	184	114	79	28	143	63

*Numbers indicate number of programs which a given parameter was available.

**Additional degree includes MS/MSc, PhD, MPH, and MBA.

ENT, otolaryngology; GEN, general surgery; IR, interventional radiology; NEUROSURG, neurosurgery; OB-GYN, obstetrics and gynecology; OPH, ophthalmology; ORTH, orthopedic surgery; PD, residency program director; PLAST, plastic and reconstructive surgery; THOR, thoracic surgery; URO, urologic surgery; VASC, vascular surgery.
